# Denaturing Gradient Gel Electrophoresis (DGGE) as a Powerful Novel Alternative for Differentiation of Epizootic ISA Virus Variants

**DOI:** 10.1371/journal.pone.0037353

**Published:** 2012-05-18

**Authors:** Marisela Carmona, Dagoberto Sepúlveda, Constanza Cárdenas, Luis Nilo, Sergio H. Marshall

**Affiliations:** 1 Laboratorio de Patologenos Acuícolas, Núcleo Biotecnología Curauma, Pontificia Universidad Católica de Valparaíso, Campus Curauma, Valparaíso, Chile; 2 Laboratorio de Genética e Inmunología Molecular, Instituto de Biología, Facultad de Ciencias, Pontificia Universidad Católica de Valparaíso, Campus Curauma, Valparaíso, Chile; 3 Núcleo Biotecnología Curauma, Pontificia Universidad Católica de Valparaíso, Valparaíso, Chile; University of Western Ontario, Canada

## Abstract

Infectious Salmon Anemia is a devastating disease critically affecting world-wide salmon production. Chile has been particularly stricken by this disease which in all cases has been directly related with its causative agent, a novel orthomyxovirus which presents specific and distinctive infective features. Among these, two molecular markers have been directly associated with pathogenicity in two of the eight RNA sub genomic coding units of the virus: an insertion hot spot region present in viral segment 5 and a Highly Polymorphic Region (HPR) located in viral segment 6. Here we report the successful adaptation of a PCR-dependent denaturing gel electrophoresis technique (DGGE), which enables differentiation of selected reported HPR epizootic variants detected in Chile. At the same time, the technique allows us to distinguish one nucleotide differences in sequences associated with the intriguing, and still not well-understood, insertion events which tend to occur on RNA Segment 5. Thus, the versatility of the technique opens new opportunities for improved understanding of the complex biology of all ISA variants as well as possible applications to other highly variable pathogens.

## Introduction

Infectious salmon anemia (ISA) is a viral disease that causes severe losses in the Atlantic salmon (*Salmo salar*) farming industry. Historically, the disease first spread rapidly along the Norwegian coast [1], then to Canada and Scotland [2]–[4], later in the Faroe Islands and eventually to the USA [5]. In Chile, ISA was first detected in marine farmed Coho salmon (*Oncorhynchus kisutch*) in June 1999 [6] and the first outbreak in *Salmo salar* occurred in 2007 [7]. The virus has also been reported to be present in apparently healthy wild animals [8].

The etiological agent, named ISA virus or ISAv [9], has been identified as a member of the *Orthomyxoviridae* family [10], [11] and constitutes the only member of the genus *Isavirus* [9]. It is an enveloped virus whose genome consists of eight negative-sense, single-stranded RNA segments [12], all of which have been fully sequenced [13]. Of the eight segments, two coding segments are considered key elements in defining pathogenicity. These are segment 5, encoding a fusion (F) protein that appears to be involved in the fusion of viral and cellular membrane and segment 6, which encodes a haemagglutinin-esterase (HE) protein that mediates both receptor-binding and receptor-destroying activities, as well as putatively participates in the fusion process [14]–[16]. Indeed, of all sub-genomic segments these two display the highest mutation rates, which were measured as 0.67×10^−3^ and 1.13×10^−3^ nucleotides per site per year for segment 5 and 6 respectively [17].

Variability can be targeted to certain regions in both segments 5 and 6. For segment 5, there is an insertion encoding between 8 and 11 amino acids located near the cleavage site of the protein. Intriguingly, the inserted sequence in segment 5 comes from either the same and/or from different viral segments. For Norwegian strains, the 8-amino acid encoding insertion, IN1, derives from segment 3, while the 11-amino acid encoding insertion, IN2, and the 10 amino acid encoding insertion, IN3, both come from segment 5 [18]. For Chilean isolates, an 11-amino acid encoding insertion has been described as IN4 and derives from segment 2, which is one of the three segments coding for the complex viral RNA polymerase [19]. Nevertheless, not all epizootic isolates detected in Chile display an insertion in segment 5 [7]. With regard to virulence, there seems to be a correlation between the potential encoding of a key amino acid in position 266 (either Q or L), upstream of the putative R267 cleavage site in the maturation process of the protein, and an insertion event [20]. On the other hand, for segment 6, the target variable region involves selective deletions inside a 35 amino acid encoding motif, eliminating from 7 to 23 residues [18], that represent a highly polymorphic region (HPR) which has turned out to be pivotal in virulence determination. Over 30 different HPRs have been reported, but HPR7b is the most frequently found in epizootic outbreaks in Chile. It is a highly virulent strain with a prevalence of 79%, which differs from all other documented HPR7's by only one amino acid [19], [21]. Variants without deletions in the HPR have been designated as HPR0. These appear to be an avirulent or asymptomatic phenotype, which does not produce a cytopathic effect *in vitro* or tissue damage *in vivo* and to date researchers have unable to grow it in tissue culture [22], [23].

Currently, ISAv diagnosis is mostly based upon PCR procedures [24]–[27]. Due to its accuracy, speed and reproducibility, qRT-PCR is the most commonly used technique [27], [28]. Despite this, qRT-PCR alone is not conclusive and other procedures are needed to confirm infection status. Among these, the indirect fluorescent antibody test (IFAT) is commonly used for *in situ* viral detection while cell-line tissue culture is used for virus infectivity, isolation and neutralization [29]–[32]. Although these techniques are well-known and powerful tools by themselves, as a result of the extreme variability displayed by ISA viral variants, robust confirmatory diagnosis is only achieved after sequencing of the DNA amplicons from the PCR test. In fish disease diagnosis, this extra step prevents the rapid response required for critical decision-making regarding the survival of affected specimens. Therefore, alternative, fast and reliable novel techniques are urgently required to contribute to the understanding and characterization of the array of viral variants arising in breeding centers; which will complement the gold standard of sequencing, by providing pre-sequencing scanning of field samples.

In light of this need, we have turned to Denaturing Gradient Gel Electrophoresis (DGGE), a molecular fingerprinting technique that has been extensively used in several areas of research to examine microbial diversity in complex communities [33]–[38]. In DGGE, polymerase chain reaction (PCR)-generated DNA fragments of the same length but with different base-pair sequences can be fully separated in a fine-tuned gradient gel. Thus, DGGE constitutes a robust procedure by which a single point mutation can be detected [33], [34]. The rationale behind it is that fine separation is based on the melting behavior of double-stranded DNA and that melting behavior in turn depends on the base-pair composition of the target DNA [35]. In practice, separation is based on the electrophoretic mobility of a partially melted double-stranded DNA molecule in resolving polyacrylamide gels. This mobility, which is decreased, compared with that of the fully helical form of the molecule. Molecules with variant DNA sequences may have different melting behavior and will therefore stop migrating at different positions in the gel. In DGGE the use of a GC clamp modifies and stabilizes the melting behavior of the DNA sequences, preventing the complete denaturation of the products and allowing the separation of the samples based in their melting profile and not in their size [33], [39]. In our case, we have adapted DGGE to variable target coding regions of the ISA virus important for pathogenicity. Specifically insertion events in a hot-spot region of a viral segment and/or the defined functionally-related deletions in another have been targeted. Hence, we are testing the adaptability of the technique to differentiation of viral variants around the insertion and flanking regions of viral segment 5 and the deletion events in the HPR coding region of segment 6. We believe that the proposed adaptation of the PCR-DGGE technique to viral research in general, and to the ISA virus in particular, has the potential to shed light on virulent as well as avirulent variants of the virus and on the effects of treatment procedures with regard to infection control. The adapted DGGE technique was used with field samples obtained from routine diagnostic analysis of ISAv variants present in Chile, with consistent and reproducible results.

Although the present work is a specific adaptation of the standard technique, focusing in characterizing virus variants and restricted to salmonid fish, it clearly demonstrates its versatility, since our works escapes from the normal allele differentiation of DGGE application. Therefore, it opens the possibility of broader scopes of adaptation applicable to other biological systems as well. In addition, once standardized the adapted procedure with well-defined standards, it can be easily applicable to a large number of samples with a high degree of confidence thus constituting an amicable and innovative procedure.

## Results and Discussion

### Design and primer selection

**Figure 1:**
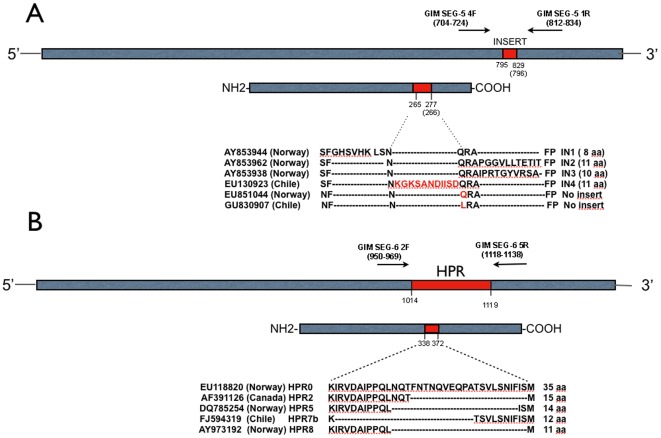
Primer locations for segments 5 (A) and 6 (B), respectively, relating nucleotide and amino acid positions against reference Norwegian and Chilean isolates. Nucleotide and amino acid number position under the scheme is based on sequences EU130923 (EU851044) for segment 5 and EU118820 for segment 6.

As primer design is a key issue for DGGE separation, the following common strategy was used to distinguish variants in segment 5 and 6. Forward and reverse putative primers were designed, chemically synthesized and evaluated *in silico*. Of all the combinations, one set was selected for each segment based on three key and distinctive features: notable differences in %GC in each amplicon, specificity and single-band resolution in regular agarose gel electrophoresis. Those selected were first analyzed in perpendicular DGGE to establish the denaturant conditions and finally the different amplicons were resolved in parallel DGGE. Figure 1 shows the location of selected primer sets for each segment and Table 1 shows the expected-size of the amplicons. For segment 5, three sequences were selected for DGGE analysis which would allow us to differentiate three variants: one with insertion; and two without insertion containing either A or U at the key 797 nucleotide Q/L 266 encoding amino acid position, expecting full resolution of these alternatives via DGGE analysis. Correspondingly, five deletion variants for segment 6 were selected for resolution, as they all correspond to natural Chilean isolates. The selected components to be used for DGGE analysis are summarized in Table 1.

**Table 1:** Selected components for DGGE analysis.

Segment	Primers	Variants	GenBank accession N°	Amplicon size (bp)
5	GIMSEG-5 4F – GIMSEG-5 1R	Insert	EU130923	164
		No insert (Q_266_)	EU851044	131
		No insert (L_266_)	GU830907	131
6	GIMSEG-6 2F – GIMSEG-6 5R	HPR0	EU118820	189
		HPR2	AF391126	129
		HPR5	DQ785254	126
		HPR7b	FJ594319	120
		HPR8	AY973192	117

### Optimizing DGGE conditions

Three pivotal conditions needed to be optimized for accurate resolution: proportion of urea/formamide as key denaturant components; acrylamide percentage as the molecular weight separation parameter and lastly, fine-tuning the primer to substrate ratio to avoid intermediate artifacts known to occur during the amplification step due to the complexity of the added GC clamp structure. The exact causes of artifacts that can lead to the generation of additional bands are unknown, but the potential causes include: incomplete DNA strand extension over the template strand, followed by switching to the complementary strand, from which DNA synthesis continued over the complementary strand [39], [40]; or the formation of heteroduplexes during the PCR reaction in which two or more homologous genes or alleles that differ for a point mutation or insertion/deletion are amplified using the same primers [41]–[43], or anomalous melting behavior due to either the dragging of fragments through the gel matrix or secondary structure formation of DNA single strands [44]

**Figure 2:**
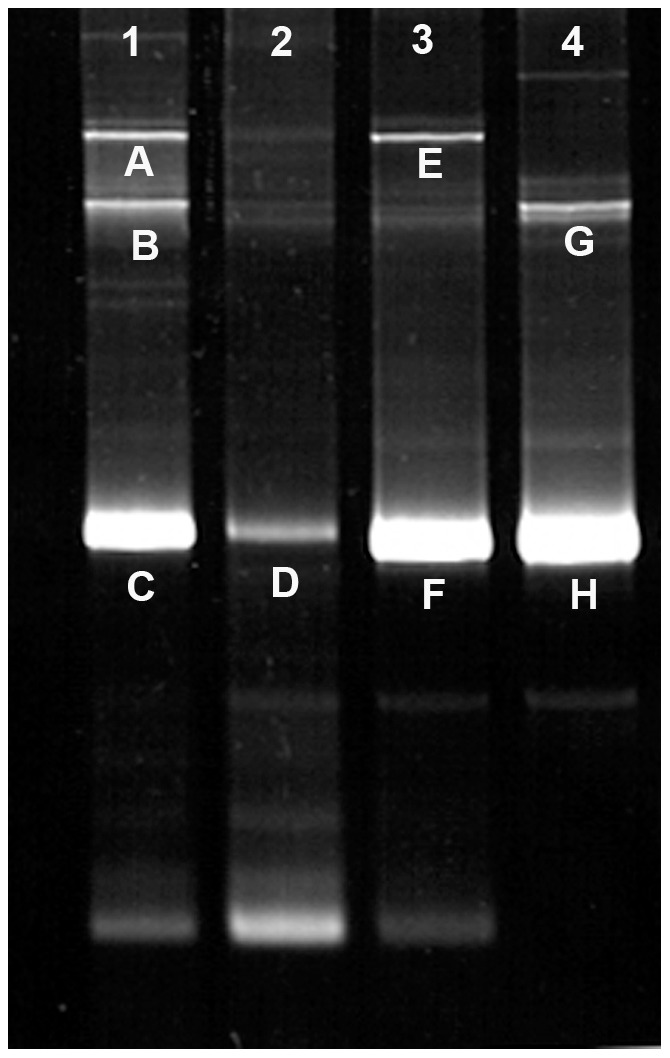
DGGE analysis of four HPR variants under non standardized DGGE separation. Lanes 1–2 two independent HPR2 isolates, lanes 3–4 two independent HPR7b isolates.

Figure 2 clearly shows that under non-standardized conditions, DGGE yields an array of specific as well as nonspecific bands or “structures” formed during the PCR amplification. In this case, four isolates corresponding to two different HPR variants were resolved. Lanes 1 and 2 correspond to two different HPR2 variants and Lanes 3 and 4 to two different HPR7b variants. Since bands C, D, F and H correspond to the expected size of the amplicons, we inferred that bands A, B, E and G were either intermediate reactions, artifacts or the coexistence of more than one HPR variant in the same sample.

**Figure 3:**
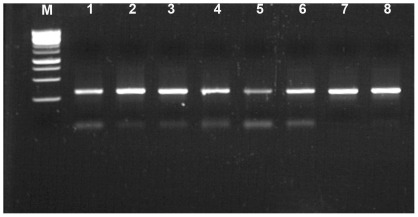
Neutral agarose gel electrophoresis of reamplified assorted DGGE-resolved products from **Figure 2**. Lane M: DNA 1-Kb marker, lanes 1–8 correspond to the bands letters (A–H) in Figure 2.

In order to resolve this situation, each band was recovered from the gel and reamplified using specific primers for each isolate, though this time without the GC clamp, and then submitted to DNA sequencing. Figure 3 shows the result of the reamplification of the eight selected bands in a neutral agarose gel. Similar sizes are expected for both HPR2 and HPR7b, since they are similar in length.

**Figure 4:**
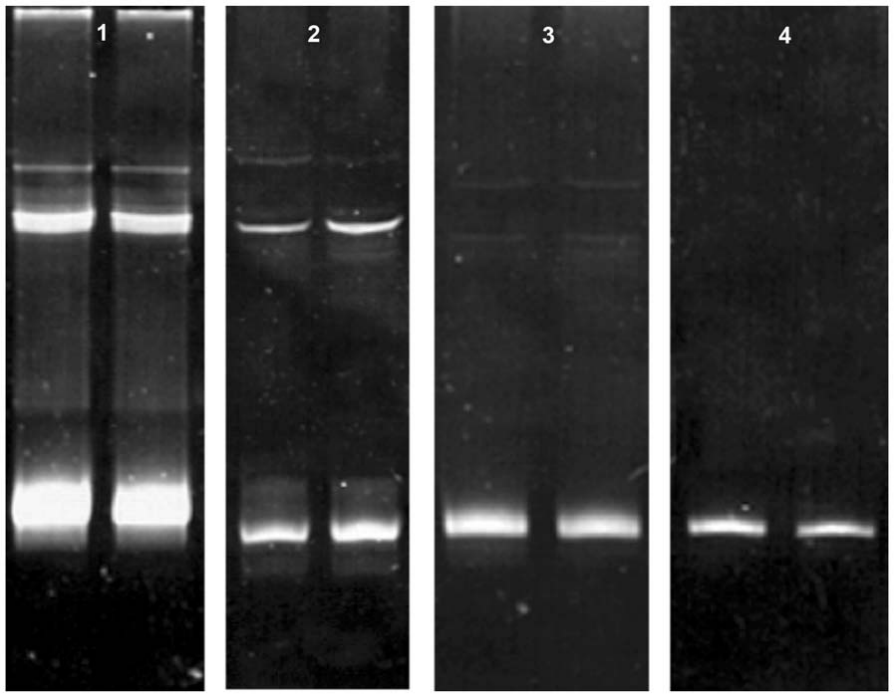
DGGE analysis for different primer concentrations of a single HPR7b variant. Duplicate reactions: Lane 1: 25 nM, lane 2: 20 nM, lane 3: 18.6 nM, lane 4: 12.5 nM.

Sequencing and BLAST [45] bioinformatics analyses confirmed that bands 1–4 and 5–8 (Figure 3) correspond to truly independent HPR2 and HPR7b variants, respectively. As a consequence, these results suggest that we were not dealing with dual infections and that in order to avoid artifacts, primer concentration appeared to be a critical and limiting component of exclusive amplification of specific sequences. Therefore, all amplifications were carried out in a range of 12.5 to 25.0 nM. Figure 4 shows that for a single HPR7b variant, a primer concentration of 12.5 nM allowed optimal resolution as a single band product.

**Figure 5:**
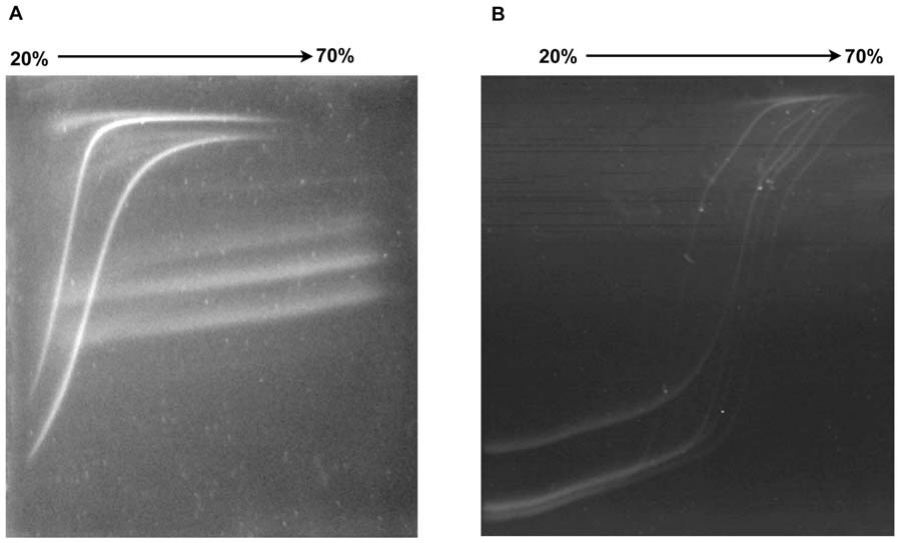
Perpendicular DGGE analyses of all selected variants for both segments. Panel A: resolution of variants in segment 5 with and without insert. Panel B: Resolution of all five HPR variants (HPR0, HPR2, HPR5, HPR7b and HPR8, correspondingly).

We subsequently made selections based on the denaturant reagent (urea/formamide) ratios. Using established DGGE protocols as reference [36]–[38] we selected 20–70% denaturants for perpendicular DGGE separation to demonstrate the partial melting conditions of the resolving samples. Figures 5A and B show the expected resolved profiles for segment 5 (with and without insertion) and for the five selected variants of segment 6. Based on these results and other confirmation experiments not shown, we decided to use a denaturant ratio of 30–60% for definitive parallel DGGE resolution conditions.

### Optimized conditions for parallel DGGE resolution

**Figure 6:**
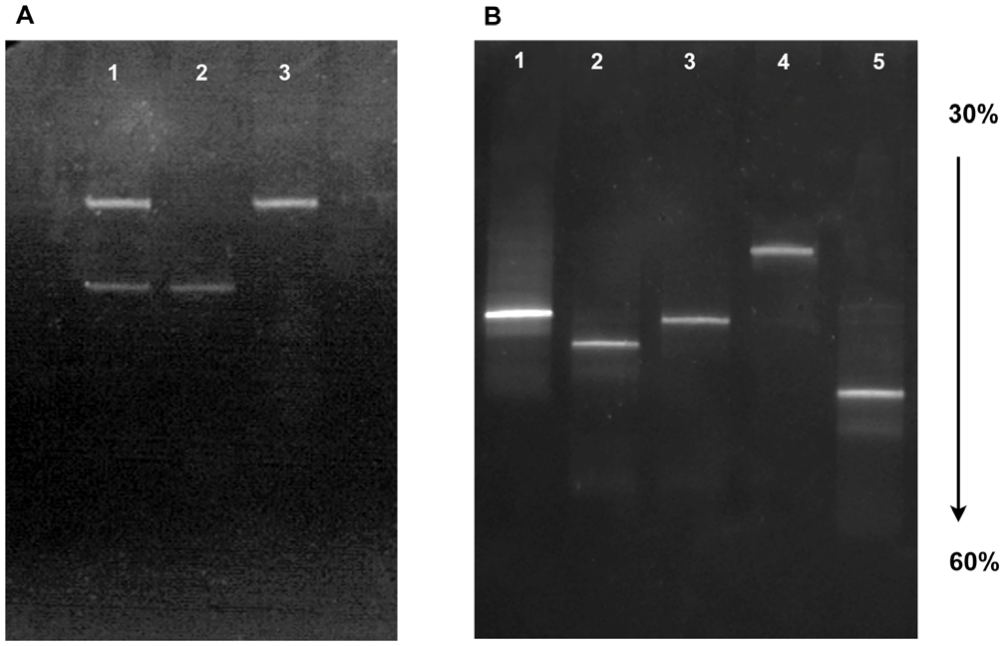
Fine resolution of ISA segment 5 and 6 of ISA variants via parallel DGGE. Panel A. Lane 1 mix of the segment 5 with and without insert, Line 2 segment 5 without insert (GU830907), Line 3 segment 5 with insert (EU130923); Panel B: Lines 1–5, HPR marker variants: HPR0, HPR2, HPR5, HPR7b and HPR8, correspondingly (for the accesion number see Table 1).

Once the primers were selected, their concentration standarized, and denaturant conditions were fully optimized, parallel DGGE resolutive gels could be run. Figure 6 shows the resolution of both segment 5 versions, with and without insertion (Panel A); and the clear resolution over the five analyzed HPR variants (Panel B).

It is clear that optimization of the primer to template ratio is a key step for accurate resolution of the amplification product. If we compare the products obtained in this Figure with that of Figure 2, we can conclude that under optimized conditions, the behavior of resolved bands is primarily based on their melting potential and not on their size.

**Figure 7:**
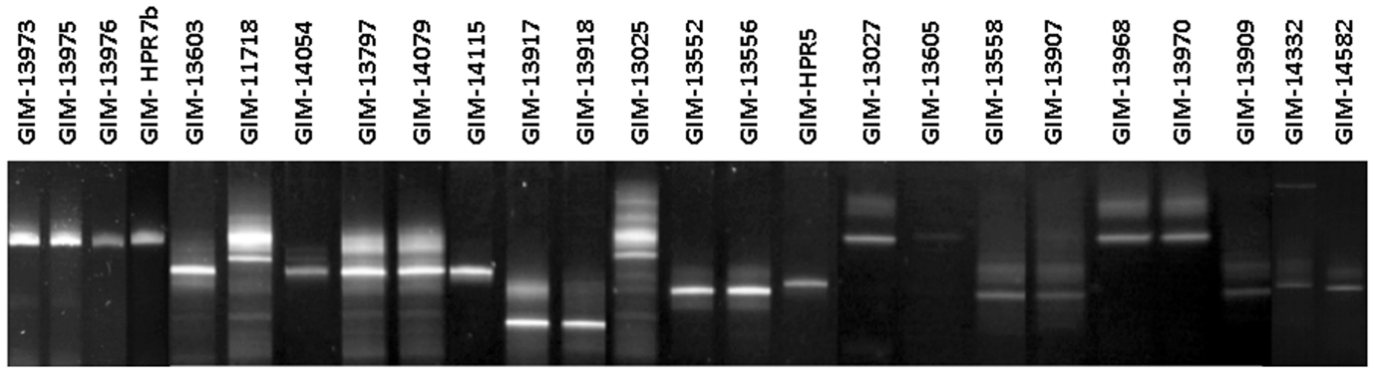
DGGE analysis of the 25 field samples showed in **Table 2**.

In order to confirm the sustainability of the standarized procedure we took 25 field random ISA isolates and in almost every case the DGGE profile coincided with the expected amplicon size and this was further confirmed by DNA sequencing (data not shown). Figure 7 shows an array of symptomatic and asymptomatic field specimens which are properly characterized by DGGE. As can be seen, field samples do show a slight background, though not significantly enough for their origins to be misinterpreted.

A sensitivity and specificity analysis was made for the 25 field samples presented in table 2. The procedure was able to distinguish between almost all the different ISAv variants of segment 6, with the exception of two samples (GIM-14332 and GIM-14582), misclassified as HPR5. These results suggest that the method has a sensitivity of 100% and a specificity of 92%, however it is most meaningful to do this analysis for each variant. Thus it was found that for variants HPR0, HPR7 and HPR8 the procedure has a sensitivity and specificity of 100%, but in the case of variants HPR2 and HPR5 misclassification can still occur, decreasing the overall specificity of the method.

**Table 2:** Field samples analyzed and corresponding variants identified by sequencing (gold standard) and by DGGE.

Sample	Sample Code	Variant Gold standard	Variant DGGE
**1**	GIM-13973	HPR7b	HPR7b
**2**	GIM-13975	HPR7b	HPR7b
**3**	GIM-13976	HPR7b	HPR7b
**4**	GIM-HPR7b	HPR7b	HPR7b
**6**	GIM-13603	HPR0	HPR0
**7**	GIM-11718	HPR7b	HPR7b
**8**	GIM-13797	HPR0	HPR0
**9**	GIM-14054	HPR0	HPR0
**10**	GIM-14079	HPR0	HPR0
**11**	GIM-14115	HPR0	HPR0
**12**	GIM-13917	HPR8	HPR8
**13**	GIM-13918	HPR8	HPR8
**14**	GIM-13025	HPR7b	HPR7b
**15**	GIM-13552	HPR2	HPR2
**16**	GIM-13556	HPR2	HPR2
**17**	GIM-HPR5	HPR5	HPR5
**18**	GIM-13027	HPR7b	HPR7b
**19**	GIM-13605	HPR7b	HPR7b
**20**	GIM-13558	HPR2	HPR2
**21**	GIM-13907	HPR2	HPR2
**22**	GIM-13968	HPR7b	HPR7b
**23**	GIM-13970	HPR7b	HPR7b
**24**	GIM-13909	HPR2	HPR2
**25**	GIM-14332	HPR2	**HPR5**
**26**	GIM-14582	HPR2	**HPR5**

**Figure 8:**
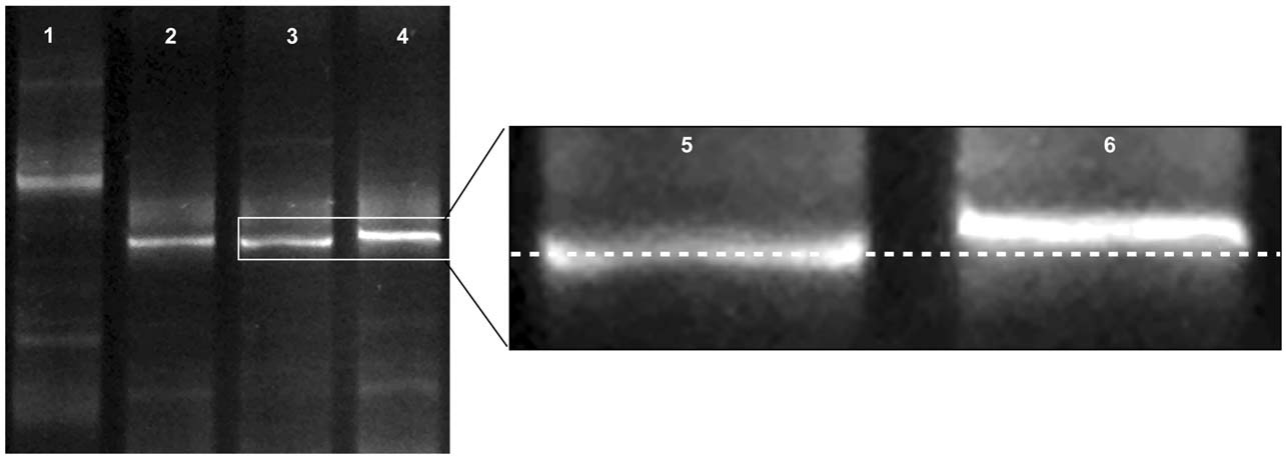
Resolution via DGGE of a single nucleitide difference in segment 5. Lane 1 segment 5 with insert, lanes 2–3 segment 5 without insert and Q_266_, and lane 4 segment 5 without insert and L_266_ (for accession numbers see Table 1).

Finally, in order to test the validity of the proposed procedure, we attempted to resolve the single nucleotide difference in position 797 in segment 5 that determines the amino acid shift of Q to L known to be correlated with pathogenicity. Figure 8 shows an interesting subtle difference between the two samples, which deserves to be experimentally expanded in order to evaluate its potential as an alternative versatile application for the procedure.

### Conclusion

The adaptation of PCR-DGGE to a viral surveillance such as the one presented in this paper in order to differentiate variants in segment 5 and 6 of ISAv, has a number of advantages over other assays. The most relevant is that it constitutes a fast, inexpensive and accurate diagnostic tool, with high sensitivity and specificity, which can also be extended to other pathogenic agents. This assay is also not technically demanding and as it is a fingerprinting technique and in many cases sequencing may be avoided when this assay has been properly validated. If so, the technique can constitute a valuable complement to field samples analysis for pre-sequencing scanning. The procedure distinguishes a difference that involves a single nucleotide change (C**A**G to C**U**G in viral segment 5, responsible for the single amino acid change (Q to L) which is presumably associated with virulence and seems to be associated with the promotion of an insertional event). In conclusion, we are offering a versatile composite technique that could become an alternative or complementary diagnostic tool for the ISA virus outbreaks in aquaculture facilities and especially if it provides the ability to detect existing and future variants. Additionally, the application of PCR-DGGE can be expanded to more basic areas of research such as attempts to understand ISAv behavior *in vivo*, particularly if related with the puzzling HPR0 genotypes. Taking advantage of the fact that the procedure shows that in some bacterial species significant differences in predominant bacterial composition are key elements to distinguish between asymptomatic and symptomatic cases, we are initiating a research line with PCR-DGGE on the ISA virus to attempt to answer the key questions of co-infection and its impact in unknown processes such as viral persistence and asymptomacy.

## Materials and Methods

### Fish samples and viral diagnosis

Pooled organs (kidney, heart and gills) from naturally infected *Salmo salar* specimens as well as pooled organ controls were provided and certified as such by SERNAPESCA, the national institute responsible for fish health control. Samples were confirmed as positive or negative to the virus in our laboratories via RT-PCR and qRT-PCR [28].

### ISA virus variants

The variants available for our study were natural isolates characterized and sequenced in our lab as follows: isolate GIM-1 containing a 33 nucleotide-long segment 5 (EU130923 Figure S1) and the genotype HPR7b (FJ594319) for segment 6; and two isolates, GIM-2 and GIM-3 (EU851044; GU830907, respectively) both lacking insert in segment 5, and the phenotypes HPR2 (AF391126) and HPR0 (EU118820) for segment 6, correspondingly. Additionally, GIM 2 and 3 differ only in one key nucleotide in position 797 (C**A**G and C**U**G) which renders one amino acid substitution (Q to L) in position 266 of the corresponding protein. We also analyzed one single specimen of segment 6 genotypes HPR5 (DQ785254) and an unknown situation for segment 5, as well as HPR8 (AY973192) and no insert and “L” for segment 5, respectively.

#### RNA extraction

Organ pools were minced and homogenized using a MagNA Lyser (Roche, USA) and total RNA was isolated by means of an RNeasy Mini Kit (Qiagen, MD, USA) for use as the source in generating cDNAs.

### Bioinformatic analysis for primer modeling and design

The RNA sequences for segments 5 and 6 of the ISAv variants were obtained from GenBank database. These were aligned to find highly conserved regions for the design of specific primers able to amplify short regions compatible in GC content with the required GC clamps needed for DGGE (Figure S1 and S2). Eventually, two sequences were selected for segment 5 (with/without insertion) and five for the different HPRs for segment 6. Prior to definitive selection of the primers, putative amplicons were analyzed for optimal size and GC content (Table S1). The Vector NTI Suite 9.0 software package [46] was used to interpret multiple alignments and manual adjustments were made with the BioEdit alignment editor [47] for definitive primer modeling.

### Primer design and modeling

Primers were designed using Primer3 software [48], and some manual adjustments were made when required. Primer properties were then calculated with OligoCalc [49]. Initially, several primers sets were selected based on sequence conservation, stringent specificity, production yield and lack of secondary structure. Based on calculations and previous experiments [50] we selected a set of primers for this study with a clamp of 30 nucleotides [51], [52] added to the 5′ terminal end (Table S2).

### Initial cDNA synthesis and primary amplification

dscDNA was obtained from the total RNA using random hexa primers driven by Superscript III reverse transcriptase (Invitrogen) in accordance with manufacturer's instructions. Primary amplifications were obtained using specific primers for both segments 5 and 6 (primers GIM SEG-5 Ext-F and GIM SEG-5 Ext-R for segment 5 and GIM SEG-6 4F and GIM SEG-6 1R for segment 6, respectively) in 12.5 µl reactions containing 2 µL of cDNA, 2 U Go Taq® Flexi DNA Polymerase (Promega, Madison, US), 1× Go Taq® Flexi buffer (Promega Corporation, Madison, US), 1 mM of MgCl_2_, and 250 µM of deoxynucleoside triphosphate (dNTP). The resultant amplicons were resolved by agarose gel electrophoresis, excised from the gel and purified using a gel extraction kit (E.Z.N.A, Omega Bio-tek).

### Secondary amplification

Diluted aliquots of purified DNA were then re-amplified using internal specific primers containing the required GC clamps for PCR-DGGE analysis. Amplification was performed in 40 µl reactions containing 2 µl DNAs, 2 U Go Taq® Flexi DNA Polymerase (Promega, Madison, US), 1× Go Taq® Flexi buffer (Promega Corporation, Madison, US), 2 mM of MgCl_2_ and 250 µM of deoxynucleoside triphosphate (dNTP). Final primer concentration ranged between 12.5 to 25 nM with one cycle of initial denaturation at 95°C for 2 min, then 35 cycles at 95°C for 30 s, followed by primer annealing at 57°C for 30 s and 1 min extension at 72°C. A final extension cycle was performed for 5 min at 72°C. Products were resolved by 1% (w/v) agarose gel electrophoresis and visualized in GelRed stained gels (PhotoCapture; DNR Bio-imaging System, Ltd. Israel).

### Denaturing Gradient Gel Electrophoresis (DGGE)

First, perpendicular gels were run to determine the melting behavior of the DNA sequences and to establish the optimal denaturing range in order to achieve selective sample resolution. The gradient of denaturants and running conditions were optimized as follows: 40 µL of GC clamped-amplicons were resolved in 8% acrylamide (37.5∶1, acrylamide∶Bis-acrylamide) perpendicular gels in a 20–70% gradient of denaturants (where 100% denaturant concentration was equal to 7 M urea (Winkler, Ltd) and 40% (v/v) of deionized formamide (Amresco® Solon Ind., Ohio). TEMED and ammonium persulfate were added to a final concentration of 0.1% each. Electrophoresis was run in 1× TAE buffer (40 mM Tris-acetate, 1 mM EDTA pH 8.0) at constant 130 V for 90 min and at 56°C using the Bio-Rad D-Code™ Universal Mutation Detection System. Gels were stained with 3× Gel Red (Biotium Inc., CA, USA) in 1× TAE buffer for 30 min and visualized as described above. Once optimized, amplicons were analyzed by parallel DGGE using a narrower range of denaturants (30–60%) under the same running conditions as described above. In the optimization process and in order to confirm band specificity, DGGE bands were excised from the original gel and incubated in 100 µl of sterile distilled water at 4°C overnight. A 10 µl aliquot of elution was used for PCR amplification of the DNA fragments. PCR products were visualized, bands excised, and purified for sequencing with a DNA gel extraction kit (E.Z.N.A, Omega Bio-tek) (Macrogen, Korea).

### Field Sample Analysis

In order to validate the technique we processed and ran, with the optimized conditions, 25 field samples provided and certified by SERNAPESCA, corresponding of different ISAv variants (Table 2). The samples were analyzed to establish the sensitivity and specificity of the technique. This analysis was performed constructing contingency tables with the DGGE results compared against the gold standard method (sequencing) [53], [54]. The analysis was made only for segment 6 variants, because in the case of segment 5, all the samples correspond to the same variant, containing the IN4.

## Supporting Information

Figure S1 **Nucleotide sequence alignment for seven isolates of segment 5.** Sequentially: four Norwegian isolates; one Chilean isolate with insert (EU130923) and two reference isolates without insert (GU830907 and EU851044).
(DOC)

Figure S2 **Nucleotide sequence alignment for HPR region of segment 6.** Sequentially: HPR0, HPR2, HPR5, HPR7b and HPR8.
(DOC)

Table S1 **%GC for segment 5 primer sets of three isolates with/without insert (Upper table) and %GC for segment 6 primer sets of five isolates (Bottom table).**
(DOC)

Table S2 **Primer sets (forward and reverse) for PCR-DGGE analysis.**
(DOC)
