# Supplementary material for: Denaturing Gradient Gel Electrophoresis (DGGE) as a Powerful Novel Alternative for Differentiation of Epizootic ISA Virus Variants
Source: PLoS One. 2012 May 18;7(5):e37353. doi: 10.1371/journal.pone.0037353 (PMC3356253; doi:10.1371/journal.pone.0037353)
Supplement: Figure S1 — Nucleotide sequence alignment for seven isolates of segment 5. Sequentially: four Norwegian isolates; one Chilean isolate with insert (EU130923) and two reference isolates without insert (GU830907 and EU851044). (DOC) [file pone.0037353.s001.doc]

**Figure S1.** Nucleotide sequence alignment for seven isolates of segment 5 sequentially: four Norwegian isolates; one Chilean isolate with insert (EU130923) and two reference isolates without insert (GU830907 and EU851044).

**598 608 618 628 638 648 658 668 678 688**

**....|....|....|....|....|....|....|....|....|....|....|....|....|....|....|....|....|....|....|....|**

IN1  **GTACTGGTATCGGCCAAATGCAATGAAATTTCGTTCAGAGTGGTGCCGTTCCATTCTGTACCAGACAGGCTAGGGTTCGCTAGAACTAGTTCTTTTACAC**

IN2 **GTACTGGTATCGGCCAAATGCAATGAAATTTCATTCAGAGTAGTGCCGTTCCATTCTGTACCAGGCAGGCTAGGGTTCGCTAGAACTAGTTCTTTTACAC**

IN3 **GTACTGGTATCGGCTAAATGCAATGAAGTTTCATTCAGAGTAGTGCCGTTCCATTCTGTACCAGACAGGCTAGGGTTCGCTAGAACTAGTTCTTTTACAC**

IN3B  **GTACTGGTATCGGCTAAATGCAATGAAGTTTCATTCAGAGTAGTGCCGTTCCATTCTGTACCAGACAGGCTAGGGTTCGCTAGAACTAGTTCTTTTACAC**

EU130923 **GTACTGGTATCGGCCAAATGCAATGAAATTTCATTCAGAGTAGTGCCGTTCCATTCTGTACCAGACAGGCTAGGGTTCGCTAGAACTAGTTCTTTTACAC**

GU830907 **GTACTGGTATCGGCCAAATGCAATGAAATTTCATTCAGAGTAGTGCCGTTCCATTCTGTACCAGACAGGCTAGGGTTCGCTAGAACTAGTTCTTTTACAC**

EU851044 **GTACTGGTATCGGCCAAATGCAATGAAATTTCATTCAGAGTAGTGCCGTTCCATTCTGTACCAGACAGGCTAGGGTTCGCTAGAACTAGTTCTTTTACAC**

**______________________ ________________________**

**GIM SEG-5 1F GIM SEG-5 3F ______________________**

**GIM SEG-5 4F**

**698 708 718 728 738 748 758 768 778 788**

**....|....|....|....|....|....|....|....|....|....|....|....|....|....|....|....|....|....|....|....|**

IN1 **TAAGAGCCAACCTCGCTAACCAGCATGGATGGTCTAAATACAGCTTCGGACACTCTGTGCACAAGCTTTCTAAC~~~~~~~~~~~~~~~~~~~~~~~~~~**

IN2 **TAAGAGCCAACCTCGCTAACCAGCATGGATGGTCTAAATACAGCTTC~~~~~~~~~~~~~~~~~~~~~~~~AAC~~~~~~~~~~~~~~~~~~~~~~~~~~**

IN3 **TAAGAGCCAGCCTCGCTAACCAGCATGGATGGTCTAAATACAGCTTC~~~~~~~~~~~~~~~~~~~~~~~~AAC~~~~~~~~~~~~~~~~~~~~~~~~~~**

IN3B  **TAAGAGCCAGCCTCGCTAACCAGCATGGATGGTCTAAATACAGCTTC~~~~~~~~~~~~~~~~~~~~~~~~AAC~~~~~~~~~~~~~~~~~~~~~~~~~~**

EU130923 **TAAGAGCCGGCCTCGCTAACCAGCATGGATGGTCTAAATACAACTTC~~~~~~~~~~~~~~~~~~~~~~~~AACAAAGGGAAATCAGCTAATGACATTAT**

GU830907 **TAAGAGCCGGCCTCGCTAACCAACATGGATGGTCTAAATACAACTTC~~~~~~~~~~~~~~~~~~~~~~~~AAC~~~~~~~~~~~~~~~~~~~~~~~~~~**

EU851044 **TAAGAGCCGGCCTCGCTAACCAACATGGATGGTCTAAATACAACTTC~~~~~~~~~~~~~~~~~~~~~~~~AAC~~~~~~~~~~~~~~~~~~~~~~~~~~**

**798 808 818 828 838 848 858**

**....|....|....|....|....|....|....|....|....|....|....|....|....|....|....|....**

IN1 **~~~~~~~CAGAGAGCA~~~~~~~~~~~~~~~~~~~~~~~~~~~~~~~~~TTCCCAGGAGAAGAGTTCATCAAATGCTGT**

IN2 **~~~~~~~CAGAGAGCTCCTGGAGGTGTACTATTGACAGAGACCATCACATTCCCAGGAGAAGAGTTCATCAAATGCTGT**

IN3  **~~~~~~~CAGAGAGCAATTCCTAGGACTGGTTATGTTAGGAGTGCA~~~TTCCCAGGAGAAGAGTTCATCAAATGCTGT**

IN3B  **~~~~~~~CATAGAGCAATTCCTAGGACTGGTTATGTTAGGAGTGCA~~~TTCCCAGGAGAAGAGTTCATCAAATGCTGT**

EU130923 **CTCCGACCAGAGAGCA~~~~~~~~~~~~~~~~~~~~~~~~~~~~~~~~~TTCCCAGGAGAAGAGTTCATCAAATGCTGT**

GU830907 **~~~~~~~CTGAGAGCA~~~~~~~~~~~~~~~~~~~~~~~~~~~~~~~~~TTCCCAGGAGAAGAGTTCATCAAATGCTGT**

EU851044 **~~~~~~~CAGAGAGCA~~~~~~~~~~~~~~~~~~~~~~~~~~~~~~~~~TTCCCAGGAGAAGAGTTCATCAAATGCTGT**

**________________________**

**GIM SEG-5 1R**
