# Supplementary material for: Denaturing Gradient Gel Electrophoresis (DGGE) as a Powerful Novel Alternative for Differentiation of Epizootic ISA Virus Variants
Source: PLoS One. 2012 May 18;7(5):e37353. doi: 10.1371/journal.pone.0037353 (PMC3356253; doi:10.1371/journal.pone.0037353)
Supplement: Figure S2 — Nucleotide sequence alignment for HPR region of segment 6. Sequentially: HPR0, HPR2, HPR5, HPR7b and HPR8. (DOC) [file pone.0037353.s002.doc]

**Figure S2.** Nucleotide sequence alignment for HPR region of segment 6 sequentially: HPR0, HPR2, HPR5, HPR7b and HPR8.

10 20 30 40 50 60 70 80 90 100

....|....|....|....|....|....|....|....|....|....|....|....|....|....|....|....|....|....|....|....|

EU118820 HPR0 **TGCCCAGACATTGACTGGAGTAGAATTGATGCTGTTTCGTGTGAATATGACAGCTGCCCTAAGATGGTTAAAGATTTTGACCAGACAAGCTTAGGTAACA**

AF391126 HPR2 **TGCCCAGACATTGACTGGAGTAGAATTGATGCTGCTTCGTGTGAATATGACAGCTGCCCTAAGATGGTTAAAGATTTTGACCAGACAAGCTTAGGTAACA**

DQ785254 HPR5 **TGCCCAGACATTGACTGGAGTAGAATTGATGCTGCTTCGTGTGAATATGACAGCTGCCCTAAGATGGTTAAAGATTTTGACCAGACAAGCTTAGGTAACA**

FJ594319 HPR7b**TGCCCAGACATTGACTGGAGTAGAATTGATGCTGCTTCGTGTGAATATGACAGCTGCCCTAAGATGGTTAAAGATTTTGACCAGACAAGCTTAGGTAACA**

AY973192 HPR8 **TGCCCAGACATTGACTGGAGTAGAATTGATGCTGCTTCGTGTGAATATGACAGCTGCCCTAAGATGGTTAAAGATTTTGACCAGACAAGCTTAGGTAACA**

**____________________**

**GIM SEG-6 4F**

110 120 130 140 150 160 170 180 190 200

....|....|....|....|....|....|....|....|....|....|....|....|....|....|....|....|....|....|....|....|

EU118820 HPR0 **CAGACACACTTATCATGAGGGAGGTAGCATTGCACAAGGAGATGATCAGTAAACTTCAGAGGAACATCACAGATGTAAAAATCAGGGTAGACGCAATCCC**

AF391126 HPR2 **CAGACACACTTATCATGAGGGAGGTAGCATTGCATAAGGAGATGATCAGAAAACGTCAGAGGAACATCACAGATGTAAAGATCAGGGTAGACGCAATCCC**

DQ785254 HPR5 **CAGACACACTTATCATGAGGGAGGTAGCATTGCATAAGGAGATGATCAGTAAACTTCAGAGGAACATCACAGATGTAAAGATCAGGGTAGACGCAATCCC**

FJ594319 HPR7b**CAGACACACTTATCATGAGGGAGGTAGCATTGCACAAGGAGATGATCAGTAAACTTCAGAGGAACATCACAGATGTAAAA~~~~~~~~~~~~~~~~~~~~**

AY973192 HPR8 **CAGACACACTTATCATGAGGGAGGTAGCATTGCACAAGGAGATGATCAGTAAACTTCAGAGGAACATCACAGATGTAAAAATCAGGGTAGACGCAATCCC**

**___________________ ________________________**

**GIM SEG-6 2F GIM SEG-6 5F**

210 220 230 240 250 260 270 280 290 300

....|....|....|....|....|....|....|....|....|....|....|....|....|....|....|....|....|....|....|....|

EU118820 HPR0 **ACCTCAGCTGAACCAAACATTCAATACAAACCAAGTGGAGCAACCTGCAACATCTGTGTTGAGCAACATCTTCATTTCTATGGGTGTAGCAGGTTTTGGG**

AF391126 HPR2 **ACCTCAGCTGAACCAAACT~~~~~~~~~~~~~~~~~~~~~~~~~~~~~~~~~~~~~~~~~~~~~~~~~~~~~~~~~~~~ATGGGTGTAGCAGGTTTTGGG**

DQ785254 HPR5 **ACCTCAGCTG~~~~~~~~~~~~~~~~~~~~~~~~~~~~~~~~~~~~~~~~~~~~~~~~~~~~~~~~~~~~~~~ATTTCTATGGGTGTAGCAGGTTTTGGG**

FJ594319 HPR7b**~~~~~~~~~~~~~~~~~~~~~~~~~~~~~~~~~~~~~~~~~~~~~~~~~ACATCTGTGTTGAGCAACATCTTCATTTCTATGGGTGTAGCAGGTTTTGGG**

AY973192 HPR8 **ACCTCAGCTG~~~~~~~~~~~~~~~~~~~~~~~~~~~~~~~~~~~~~~~~~~~~~~~~~~~~~~~~~~~~~~~~~~~~~~~~GGTGTAGCAGGTTTTGGG**

**_________________**

**GIM SEG-6 5R**

310 320 330 340 350 360 370 380 390 400

....|....|....|....|....|....|....|....|....|....|....|....|....|....|....|....|....|....|....|....|

EU118820 HPR0 **ATTGCTCTGTTCCTAGCAGGTTGGAAGGCATGTATTTGGATTGCAGCATTCATGTACAAGTCTAGAGGTAGAATTCCACCATCGAGCCTGTCTGTTGCTT**

AF391126 HPR2 **ATTGCTCTGTTCCTAGCAGGTTGGAAAGCATGTATTTGGATTGCAGCATTCATGTACAAGTCTAGAGGTAGAATTCCACCATCGAGCCTGTCTGTTGCTG**

DQ785254 HPR5 **ATTGCTCTGTTCCTAGCAGGTTGGAAAGCATGTATTTGGATTGCAGCATTCATGTACAAGTCTAGAGGTAGAATTCCACCATCGAACCTGTCTGTTGCTT**

FJ594319 HPR7b**ATTGCTCTGTTCCTAGCAGGTTGGAAGGCATGTATTTGGATTGCAGCATTCATGTACAAGTCTAGAGGTAGAATTCCACCATCGAACCTGTTTGTTGCTT**

AY973192 HPR8 **ATTGCTCTGTTCCTAGCAGGTTGGAAGGCATGTATTTGGATTGCAGCATTCATGTACAAGTCTAGAGGTAGAATTCCACCATCGAACCTGTCTGTTGCTT**

**____________________**

**GIM SEG-6 4R**

**_____________________**

**GIM SEG- 2R**
