# Supplementary material for: Denaturing Gradient Gel Electrophoresis (DGGE) as a Powerful Novel Alternative for Differentiation of Epizootic ISA Virus Variants
Source: PLoS One. 2012 May 18;7(5):e37353. doi: 10.1371/journal.pone.0037353 (PMC3356253; doi:10.1371/journal.pone.0037353)
Supplement: Table S1 — %GC for segment 5 primer sets of three isolates with/without insert (Upper table) and %GC for segment 6 primer sets of five isolates (Bottom table). (DOC) [file pone.0037353.s003.doc]

**Table S1.** %GC for segment 5 primer sets of three isolates with/without insert (Upper table) and %GC for segment 6 primer sets of five isolates (Bottom Table).

| **Primer** | **Insert** | **No insert Q_266_** | **No insert L_266_** |
| --- | --- | --- | --- |
| GIM SEG-5 1F- GIM SEG-.5 1R | 45 | 47 | 47 |
| GIM SEG-5 3F- GIM SEG-5 1R | 44 | 45 | 45 |
| GIM SEG-5 4F- GIM SEG-5 1R | 45 | 46 | 46 |
| GIM SEG-5 1F- GIM SEG-5 2R | 46 | 47 | 47 |
| GIM SEG-5 3F- GIM SEG-5 2R | 45 | 46 | 46 |
| GIM SEG-5 4F- GIM SEG-5 2R | 46 | 46 | 46 |

| **Primer** | **HPR0** | **HPR2** | **HPR5** | **HPR7b** | **HPR8** |
| --- | --- | --- | --- | --- | --- |
| GIM SEG-6 2F- GIM SEG6 2R | 45 | 46 | 45 | 43 | 46 |
| GIM SEG-6 2F- GIM SEG6 4R | 45 | 46 | 45 | 43 | 46 |
| GIM SEG-6 2F- GIM SEG6 5R | 44 | 47 | 45 | 42 | 47 |
| GIM SEG-6 4F- GIM SEG6 2R | 44 | 45 | 44 | 43 | 45 |
| GIM SEG-6 4F- GIM SEG6 4R | 44 | 45 | 44 | 43 | 45 |
| GIM SEG-6 4F- GIM SEG6 5R | 42 | 45 | 44 | 42 | 45 |
| GIM SEG-6 5F- GIM SEG6 2R | 44 | 46 | 44 | 41 | 45 |
| GIM SEG-6 5F- GIM SEG6 4R | 44 | 46 | 44 | 41 | 45 |
| GIM SEG-6 5F- GIM SEG6 5R | 43 | 46 | 43 | 38 | 45 |
| GIM SEG-6 5F- GIM SEG6 5R | 43 | 46 | 43 | 38 | 45 |
