# Supplementary material for: Denaturing Gradient Gel Electrophoresis (DGGE) as a Powerful Novel Alternative for Differentiation of Epizootic ISA Virus Variants
Source: PLoS One. 2012 May 18;7(5):e37353. doi: 10.1371/journal.pone.0037353 (PMC3356253; doi:10.1371/journal.pone.0037353)
Supplement: Table S2 — Primer sets (forward and reverse) for PCR-DGGE analysis. (DOC) [file pone.0037353.s004.doc]

**Table S2** Primer sets (forward and reverse) for PCR-DGGE analysis.

| **Primer** | **Sequence (5 ® 3)** | **Segment** |
| --- | --- | --- |
| GIM SEG-5 Ext-F | TACAACGGAAAGGATTAAGACTG | 5 |
| GIM SEG-5 Ext-R | TCTCCTTCTAGCAGCAGGTTC | 5 |
| GIM SEG-5 1F ^(1)^ | GTACTGGTATCGGCCAAATGC | 5 |
| GIM SEG-5 3F ^(1)^ | TGCCGTTCCATTCTGTACCAG | 5 |
| GIM SEG-5 4F ^(1)^ | TACCAGACAGGCTAGGGTTC | 5 |
| GIM SEG-5 1R | ACAGCATTTGATGAACTCTTCTC | 5 |
| GIM SEG-5 2R | TCTCCTTCTAGCAGCAGGTTC | 5 |
| GIM SEG-6 2F^(1)^ | TGAGGGAGGTAGCATTGCAT | 6 |
| GIM SEG-6 4R | AGACAGGTTCGATGGTGGAA | 6 |
| GIM SEG-6 4F^(1)^ | GCCCAGACATTGACTGGAGTA G | 6 |
| GIM SEG-6 1R | CTCTAGACTTGTACATGAATGCTG | 6 |
| GIM SEG-6 2R | AAGCAACAGACAGGCTCGAT | 6 |
| GIM SEG-6 5F^(1)^ | ATCAGTAAACTTCAGAGGAACATC | 6 |
| GIM SEG-6 5R | CAATCCCAAAACCTGCTACAC | 6 |
| GC-Clamp^(2)^ | GCCGCGCCCCGCGCCCGTCCCGCCGCCCCC |  |

^(1)^ Indicates a GC clamp was attached to the 5' end of this primer.

^(2)^ GC clamp (30 bp) modified from Myers’ (1985).
